# Fabrication of an Efficient Planar Organic-Silicon Hybrid Solar Cell with a 150 nm Thick Film of PEDOT: PSS

**DOI:** 10.3390/mi10100648

**Published:** 2019-09-26

**Authors:** Sami Iqbal, Dan Su, Yi Yang, Fahim Ullah, Huanli Zhou, Azam Hussain, Tong Zhang

**Affiliations:** 1 Joint International Research Laboratory of Information Display and Visualization, School of Electronic Science and Engineering, Southeast University, Nanjing 210096, China; samiiqbal@seu.edu.cn (S.I.); yangyi19900423@sina.com (Y.Y.); fahimullah320@seu.edu.cn (F.U.); Huanli_zhou@163.com (H.Z.); Malik.azamhussain@yahoo.com (A.H.); 2 Suzhou Key Laboratory of Metal Nano-Optoelectronic Technology, Suzhou Research Institute of Southeast University, Suzhou 215123, China; jssysls@163.com; 3 Key Laboratory of Micro-Inertial Instrument and Advanced Navigation Technology, Ministry of Education, and School of Instrument Science and Engineering, Southeast University, Nanjing 210096, China

**Keywords:** hybrid solar cells, conducting polymers, ethylene glycol, single junction solar cells, cost effective

## Abstract

Organic–inorganic hybrid solar cells composed of p-type conducting polymer poly (3,4-ethylene-dioxythiophene): polystyrenesulfonate (PEDOT: PSS) and n-type silicon (Si) have gained considerable interest in recent years. From this viewpoint, we present an efficient hybrid solar cell based on PEDOT: PSS and the planar Si substrate (1 0 0) with the simplest and cost-effective experimental procedures. We study and optimize the thickness of the PEDOT: PSS film to improve the overall performance of the device. We also study the effect of ethylene glycol (EG) by employing a different wt % as a solvent in the PEDOT: PSS to improve the device’s performance. Silver (Ag) was deposited by electron beam evaporation as the front and rear contacts for the solar cell device. The whole fabrication process was completed in less than three hours. A power conversion efficiency (PCE) of 5.1%, an open circuit voltage (Voc) of 598 mV, and a fill factor (FF) of 58% were achieved.

## 1. Introduction

Recently photovoltaics have been of great interest to researchers for the application of renewable energy considering climate change, the availability of fossil fuels, and related concerns about the environment [1]. Photovoltaics are a possible quick remedy for most of these concerns, where the fabrication time of photovoltaic devices, the cost of materials, and the complexity of the related processes are the key parameters to be considered. Organic-silicon hybrid solar cells have gained significant attention due to their low cost and easy and less complex fabrication process. Currently silicon photovoltaic technologies dominate the market with over an 80% share owing to their high efficiency and reliability [2]. Silicon solar cells, however, are costly in terms of their wafers’ purification, and fabrication with sophisticated and high energy-consuming processes makes up 75% of the total cost [3]. However, the silicon photovoltaic industry is transitioning to several new technologies like thin film or thin wafer technologies to cut down material costs [4], but the choices for decreasing the fabrication cost significantly are limited. In recent times, hybrid organic-silicon solar cells have offered a concrete solution to reduce manufacturing costs by adopting less sophisticated, room temperature, scalable, and solution processed conjugated polymers to form a heterojunction with silicon at the interface [5,6,7,8]. So far, most of the organic-silicon hybrid solar cell devices with high conversion efficiency have been reported to use a textured Si substrate surface [9]. However, the planar single junction hybrid solar cell device possesses a significant gain over textured Si substrates in terms of the cost, complex processes of etching, and time consumption required for the surface texturing of Si. With the improvement in experimental processes and the study of novel carrier selective functional-materials, such as molybdenum oxide (MoO_x_) [10,11] poly (3,4-ethylenedioxythiophene): poly(styrenesulfonate) (PEDOT: PSS) [12,13] and poly-3-hexylthiophene/(6,6)-phenyl-C61-butyric acid methyl ester (P3HT/PCBM) [14,15], hybrid heterojunction solar cells could produce the next generation highly efficient organic-silicon photovoltaics with simple device architectures [16]. Different doping techniques for n-Si have also been reported in the literature for a planar n-Si/PEDOT: PSS hybrid solar cell device 13.8 with an optimized bandgap for PEDOT: PSS [17]. Some have reported different surfactants like Triton-X10 and fluorosurfactant (TX, FS) in the co-solvent for a planar hybrid solar cell device and achieved good results [18]. The technologies developed have been focused on merging the advantages of different material systems, such as carrier transport and interfacial layers, to provide higher efficiency and a lower overall fabrication cost [19]. The basic hybrid organic-silicon solar cell has a simple device structure, which is usually composed of p-type organic material PEDOT: PSS and n-type crystalline silicon. The power conversion efficiency (PCE) is basically resolute according to the optical absorption in the n-type silicon, the carrier transportation related to organic material and contact properties [2,20] as well as the effect of series resistance *R_s_* and the mechanisms of carrier recombination [21,22]. This is essential to elucidate the source of variance and enhance the attainable PCE of the planar hybrid organic-silicon solar cell [20] as a reference device before exploiting the advantages of random pyramid (RP) [6,23], silicon nanowires (SiNWs) [1,24], silicon nanoholes (SiNHs) [25], and the incorporation of metallic nanoparticles (NPs) [26] in such devices that can properly be quantified.

There are many factors that can cause variation in PCE, such as the conductivity of PEDOT: PSS, the ability of the surfactant to enhance the wettability of PEDOT: PSS, the annealing time and temperature of PEDOT: PSS, the thickness of the interfacial oxide, and the front and rear electrode arrangements [27]. It is important to understand these parameters to clarify the causes behind inconsistency in the PCEs, which can eventually assist in the fabrication of highly efficient hybrid solar cells. In this work, we demonstrated a single junction planar organic-silicon (PEDOT: PSS/Si) hybrid solar cell. We studied the external quantum efficiency (EQE) and the surface morphology of PEDOT: PSS on the planar silicon surface after being treated and cleaned by hydrofluoric acid (HF), and the power convergence efficiency (PCE) of the solar cell devices.

## 2. Experiments

To study the properties of planar organic-silicon hybrid solar cells, we fabricated an organic-silicon hybrid solar cell device utilizing an n-type Czochralski (CZ) crystalline silicon substrate with a crystallographic structure <1 0 0>, having an area of (20 mm × 20 mm) and a thickness of 200 ± 10 µm. Silicon substrates were cleaned by the RCA method employing isopropyl alcohol (IPA), Acetone, and deionized water (DI) for 10 min each at 75 °C [28]. Afterwards, the native oxide (SiO_x_) was removed via 5 wt % of hydrofluoric acid (HF) for 1 min [29,30]. The substrate was then dehydrated with pressurized nitrogen and dried for 10 min on a hotplate in an ambient environment. The organic solution was prepared by exercising PEDOT: PSS (PH1000 by Clevios, Heraeus, Hanau, Germany) mixed with 7 wt % ethylene glycol (EG) [31,32] and 0.50 wt % Triton-100 (Sigma-Aldrich, St. Louis, MO, USA) [9,33] to improve its hydrophilicity and was spin coated on the planar silicon substrate surface at 500 and 1000 rpm for 5 and 40 s, achieving a thickness of ~150 nm. The substrates were then annealed for 10 min at 120 °C on a hotplate to eliminate any solvents in the solution to form a highly conductive p-type organic thin film [34]. The electron bean evaporation (ebeam) was used to deposit silver (Ag) as the front and rear electrodes. The thickness of the front and rear electrodes was kept at 300 nm and 700 nm, respectively. The substrate temperature was kept at 25 °C during the electron bean evaporation.

In order to examine the performance and photovoltaic parameters of the as-fabricated hybrid solar cell device, we tested the samples by scanning electron microscope (SEM), SEM (X-Max, Zeiss, Ultra Plus, Gemini) by Oxford Instruments Co. Ltd. (Abingdon, UK). The front surface optical reflectance was measured by an IdeaOptics (PG2000-Pro EX) Scientific Class optical fiber Spectrometer (IdeaOptics Instruments, Shanghai, China). The Atomic Force Microscopy (AFM) topographic images were performed by a BRUKER, NanoScope^®^ V and a Nikon Multimode ScanAsyst (Billerica, MA, USA). The *J*-*V* curves were determined by a Solar Simulator Air Mass (AM) 1.5G spectrum, 100 watts (IVtest station 6000AAA) by Crowntech, Inc. (Macungie, PA, USA). The external quantum efficiency (EQE) was determined by a Qtest station 2000AD by Crowntech, Inc.

## 3. Results and Discussions

**Figure 1:**
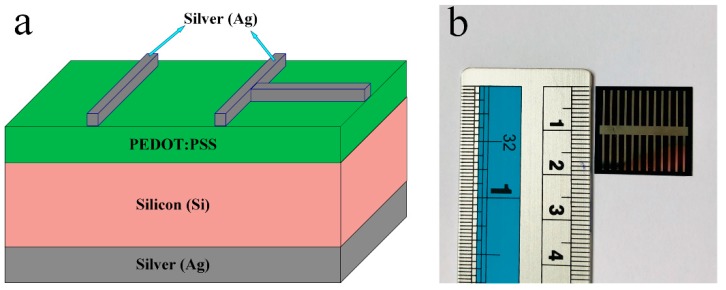
(**a**) The device configuration of the n-Si/poly (3,4-ethylenedioxythiophene): poly(styrenesulfonate) (n-Si/PEDOT: PSS) hybrid solar cell; (**b**) an image of the actual hybrid solar cell.

Figure 1a illustrates the schematic structure of the Si/PEDOT: PSS HSC, indicating the Si planar substrate, the top layer of the PEDOT: PSS, and the front and rear Ag electrodes. However, the schematic diagram does not represent the actual parameters of the solar cell device. Figure 1b displays the actual top view image of the organic silicon hybrid solar cell. All the applicable photovoltaic properties of the n-Si/PEDOT: PSS solar cells that have been obtained through illumination of the AM 1.5G solar spectrum are labelled in Table 1. As can be seen, the open circuit voltage (*V*_oc_) and fill factor (FF) have been greatly improved, whereas the short circuit current (*J*_sc_) is comparatively lower which will be discuss in a later section. The solar cell device achieved a power convergence efficiency of 5.1%.

**Figure 2:**
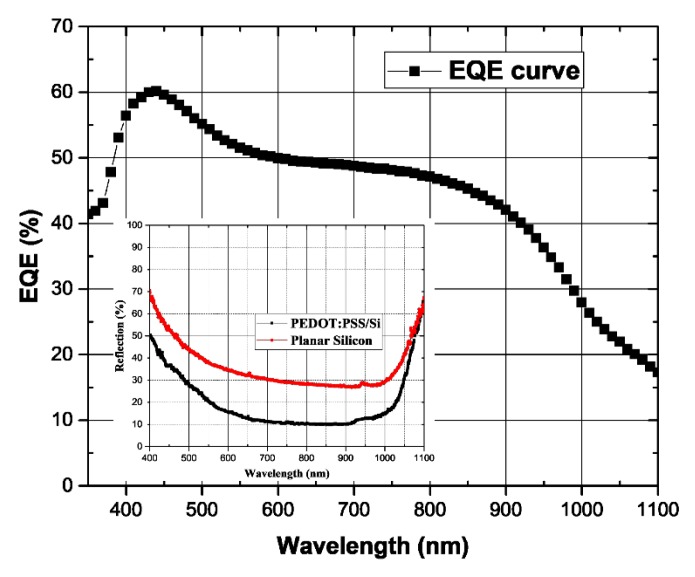
The external quantum efficiency (EQE) spectrum of the HSC device. The inset on the left display reflectance spectra of the planar Si substrate before and after spin coating of the PEDOT: PSS.

Figure 2 shows the External Quantum Efficiency (EQE) spectrum of the fabricated solar cell device with a broad EQE spectrum over 350–1100 nm. The highest achieved EQE value is 60.1% at 440 nm within the solar spectrum, while the inset graph provides the reflectance spectra of the planar Si substrate for pre and post spin coating of the PEDOT: PSS thin film, which clearly indicates that the thin film of PEDOT: PSS also serves as an antireflection coating that achieves the lowest reflectance value (9.9%) at 825 nm of the solar spectrum.

**Figure 3:**
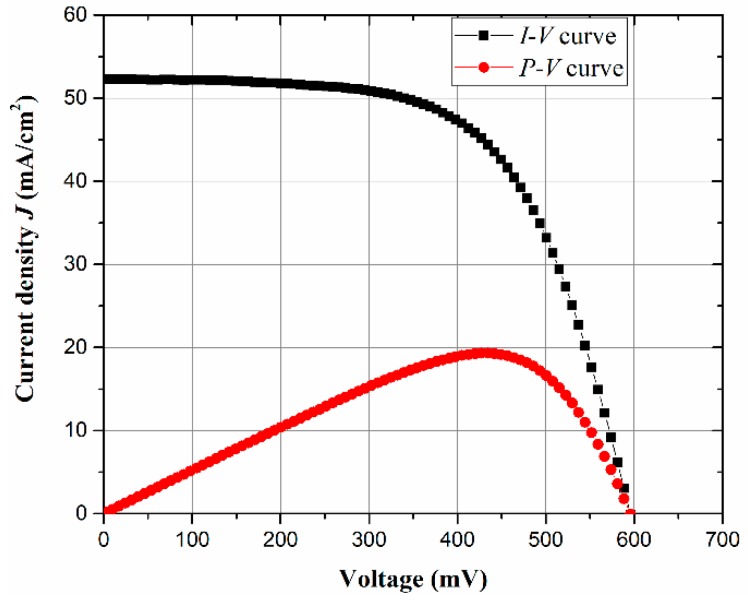
Shows the *I-V* and *P-V* curves extracted during the illumination of the AM1.5 solar simulator.

Figure 3 shows the current density-voltage (black) and power-voltage (red) curves for the PEDOT: PSS/Si single-junction hybrid solar cells with EG 7 wt %. The achieved *V*_oc_ is 598 mV, which is a good result for a planar single junction organic/Si device. However, the *J*_sc_ is 15.7 mA. The maximum achieved power point is 440 mV with an output power of 20 mW.

**Figure 4:**
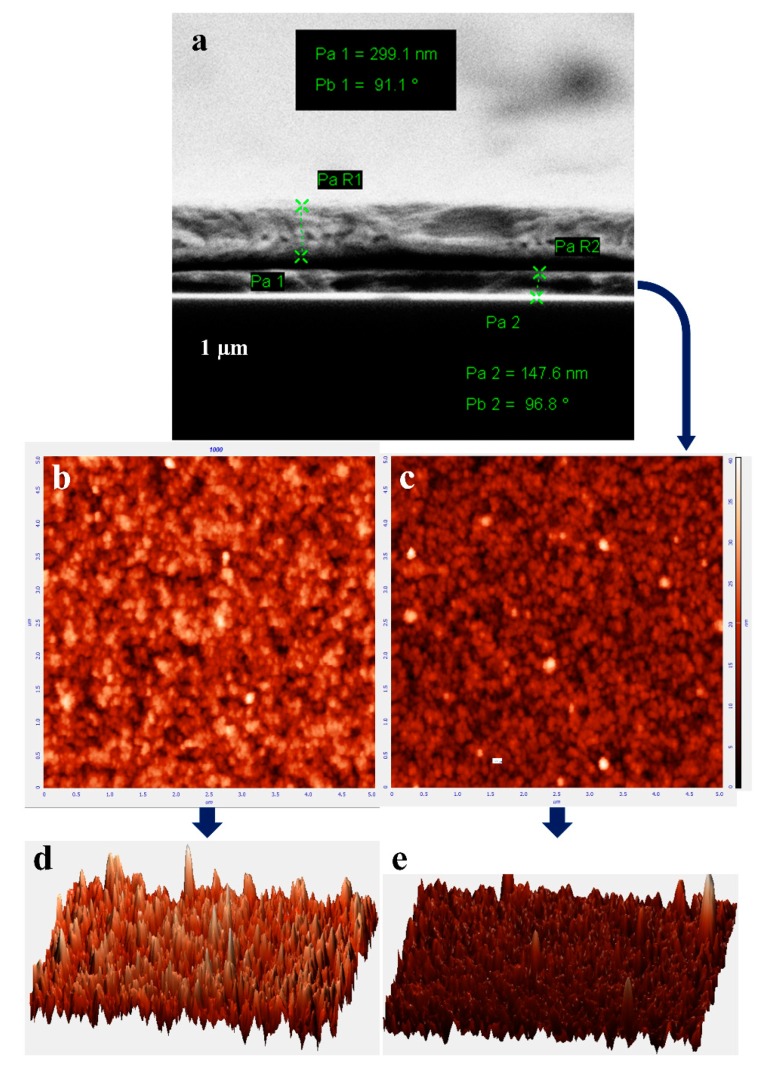
(**a**) A cross sectional SEM image of the hybrid solar cell device, (**b**) showing the AFM topographic image of the PEDOT: PSS film with 3wt % EG and (**c**) 7 wt % of EG on the Planar Si substrate; (**d**) and (**e**) illustrate the AFM 3D topography of the PEDOT: PSS films.

Figure 4a displays a cross-section SEM image of the hybrid solar cell. The device architecture can be clearly observed as the top layer of Ag (front electrode) with a thickness of 299 nm and the PEDOT: PSS sandwich between the front electrode and Planar Si substrate with a thickness of almost 148 nm, whereas the deposited thickness of the rear Ag electrode was 700 nm, which is helpful in providing smooth drift to the majority of carriers [35,36]. The *J*_sc_ of the device with a 150 nm film of PEDOT: PSS in Table 1, which is 15.7 which is lower than that of solar cell device with a 120 nm film, could be caused by the increased sheet resistance of the PEDOT: PSS film by triggering a higher resistance to the charge collection and transportation. Moreover, we have not used interfacial materials between the PEDOT: PSS and Si substrate, so the lifetime of the majority and minority carriers could be decreased at the junction region. The inset in Figure 4b shows the atomic force microscopy (AFM) topography of the spin-coated PEDOT: PSS with 3 wt % EG, and Figure 4c shows the atomic force of 7 wt % EG on top of the Si substrate. The AFM images show that the PEDOT nanocrystals are closely packed with a uniform order, showing an average roughness (Sa) of 4.38 nm with 3 wt % EG and 2.79 of Sa with 7 wt % EG. Figure 4d,e displays the AFM 3D topography of PEDOT: PSS, from which it can be clearly seen that surface morphology was better for the PEDOT: PSS with an EG solvent of 7 wt % as compared to that with an EG of 3 wt %.

## 4. Conclusion and Outlook

In summary, we fabricated a planar single-junction n-Si/PEDOT: PSS hybrid solar cell with an increased thickness of 150 nm for the PEDOT: PSS layer. The front and rear electrodes were deposited by electron beam evaporation with a thickness of 300 nm and 700 nm, respectively. We achieved a high open-circuit voltage *V*_oc_ of 598 mV, short circuit current of *J*_sc_ of 15.7 mA/cm^2^ and a fill factor FF of 58%. The maximum power convergence efficiency PCE yielded up to 5.1%. We studied the effect of the thickness of the PEDOT: PSS with different concentrations of the co-solvent EG. We found that *V*_oc_, FF, and PCE-(η) show dramatic improvement by increasing the thickness of PEDOT: PSS. However, *J*_sc_ shows a decreasing tendency with an increased thickness of PEDOT: PSS. We observed that the main reason for low *J*_sc_ (15.7) could be the elevated sheet resistance staged by the increased thickness of the PEDOT: PSS film obstructing the charge collection and transportation on the top layer, thereby making it difficult for minority carriers to reach the top Ag grid (front electrode), as well as the observation of the hole and the electron drift. The hole and electron drift mechanism can be improved by introducing a suitable and efficient interfacial layer material at the junction region between the PEDOT: PSS and Si substrate. Adding a co-solvent of EG 7 wt % made the PEDOT: PSS nanocrystal congested and stuffy with a uniform distribution order on the surface of the planar Si substrate.

## Figures and Tables

**Table 1:** The photovoltaic parameters of the n-Si/poly (3,4-ethylenedioxythiophene): poly(styrenesulfonate) (n-Si/PEDOT: PSS) hybrid solar cell with a different thickness of PEDOT: PSS and concentration of the solvent EG.

Thickness of PEDOT:PSS Film (nm)	*J*_sc_ (mA/cm^2^)	*V*_oc_ (mV)	FF (%)	PCE-η (%)	Ethylene Glycol (EG) (%)
80	13.5	418	35	1.98	3
100	14	515	41	2.9	5
120	16.5	506	38	3.2	7
150	15.7	598	58	5.1	7
